# Cardiopulmonary Exercise Testing in Elderly Patients with Cardiopulmonary Comorbidities: Safety and Clinical Feasibility

**DOI:** 10.3390/jcm15134896

**Published:** 2026-06-24

**Authors:** Miraç Öz Kahya, Mursal Isgenderli, Ömer Faruk Tüten, Öznur Yıldız

**Affiliations:** Department of Chest Diseases, Faculty of Medicine, Ankara University, 06620 Ankara, Türkiye; murselisgenderli951@gmail.com (M.I.); omertuten@gmail.com (Ö.F.T.); oznuryildiz1411@yahoo.com (Ö.Y.)

**Keywords:** cardiopulmonary exercise testing, CPET, elderly patients, safety, feasibility, pulmonary function, comorbidity

## Abstract

**Background/Objectives**: Cardiopulmonary exercise testing (CPET) provides an integrated assessment of cardiovascular, respiratory, and metabolic responses during exercise. Although CPET is increasingly used in older adults for preoperative physiological evaluation and assessment of exercise limitation, evidence regarding its safety and clinical feasibility in elderly patients with mixed cardiopulmonary comorbidities remains limited. **Methods**: In this retrospective observational study, we evaluated 235 consecutive patients who underwent CPET at a tertiary referral center. Patients were categorized into two groups according to age: ≥65 years and <65 years. Clinical characteristics, pulmonary function parameters, CPET findings, feasibility outcomes, and adverse events during testing were analyzed. **Results**: A total of 235 patients were included, with a mean age of 62.3 ± 12.8 years. Among them, 112 (47.6%) patients were aged ≥65 years and 35 (14.8%) were aged ≥75 years. Comorbidities were present in 170 patients, with hypertension being the most common. The leading indication for CPET was preoperative evaluation prior to thoracic surgery. Most elderly patients successfully completed CPET and provided clinically interpretable physiological data. In the ≥65 years group, CPET was terminated prematurely in 10 patients due to syncope, severe dyspnea, bronchospasm, chest pain, or arrhythmia. In the ≥65 years group, exercise-induced desaturation occurred in 24 patients; the lowest recorded oxygen saturation was 84%, and no desaturation episode required premature termination of the test. No major complications, deaths, myocardial infarctions, or cardiac arrests were observed during CPET or within the subsequent three days. No statistically significant differences in adverse event rates were observed between the age groups. Univariate logistic regression analysis demonstrated that lower FEV1 % predicted and lower FEV1/FVC % predicted ratio were associated with clinically significant adverse events in elderly patients [OR (95% CI): 0.96 (0.94–0.99), *p* = 0.02, OR (95% CI): 0.90 (0.84–0.96), *p* = 0.001, respectively]. **Conclusions**: CPET was feasible in the majority of elderly patients with cardiopulmonary comorbidities, with most individuals successfully completing testing and providing clinically interpretable physiological data. No major complications were observed in this cohort. These findings suggest that, when performed under appropriate supervision and careful patient selection, CPET may represent a practical tool for functional assessment and preoperative physiological evaluation in older adults. Larger prospective multicenter studies are warranted to further define its safety and feasibility in this population.

## 1. Introduction

Cardiopulmonary exercise testing (CPET) is used to evaluate exercise performance, functional capacity, and exercise-related limitations by objectively assessing symptoms such as unexplained dyspnea and fatigue. The test is performed on a treadmill or cycle ergometer using individualized protocols with progressively increasing workload tailored to the patient’s functional status [1].

The growing proportion of older adults worldwide has led to a substantial increase in the prevalence of multimorbidity, particularly cardiopulmonary diseases that impair exercise capacity and functional status [2]. Elderly patients are frequently referred for preoperative physiological assessment prior to thoracic and cardiac surgery, as well as for the evaluation of unexplained dyspnea and exercise intolerance [3,4].

CPET is commonly incorporated into preoperative physiological assessment and may provide objective information regarding functional capacity before surgery. In elderly patients, who often have multiple comorbidities and reduced physiological reserve, this information may be particularly valuable during preoperative evaluation.

Furthermore, CPET-derived parameters have been shown to provide prognostic information beyond conventional pulmonary function testing alone [3].

However, despite the potential clinical value of CPET in these settings, concerns regarding frailty, multiple comorbidities, reduced physiological reserve, and the risk of exercise-related adverse events often limit its use in older individuals. Consequently, establishing the safety and feasibility of CPET in elderly patients has become an increasingly important clinical issue [5,6].

CPET provides an integrated assessment of cardiovascular, pulmonary, and skeletal muscle function through the measurement of ventilatory and gas exchange parameters, heart rate, electrocardiography, and blood pressure during exercise. It is widely used in the clinical management of patients undergoing lung and cardiac surgery, as well as in those with chronic respiratory and cardiovascular diseases, including COPD, asthma, pulmonary hypertension, and interstitial lung disease. In addition, CPET serves as a valuable tool for exercise prescription and performance evaluation in athletes. By directly measuring maximal oxygen uptake (VO_2_max), CPET defines the physiological limits of the cardiopulmonary system and allows accurate evaluation of functional capacity, diagnosis, and monitoring of disease, as well as assessment of therapeutic response. Furthermore, exercise tolerance determined by CPET is an important clinical outcome and a well-established predictor of mortality in chronic cardiopulmonary conditions [5].

While maximal exercise testing is considered the reference standard for evaluating aerobic capacity, its applicability may be constrained in individuals with neurologic, orthopedic, or other conditions that limit the ability to achieve maximal effort [7]. Conventionally, maximal exertion is defined by reaching at least 85% of the age-predicted maximal heart rate or attaining a respiratory exchange ratio (RER) ≥ 1.10, both indicative of near-peak physiological effort. In cases where maximal testing is not feasible, submaximal CPET represents a practical alternative for estimating maximal heart rate and VO_2_max [5,7,8,9].

In addition to safety, the feasibility of CPET is an important consideration in elderly patients [10]. Feasibility encompasses the ability to successfully complete testing, achieve an adequate level of exercise effort, tolerate the procedure, and obtain clinically interpretable physiological data. These factors are particularly relevant in older adults, in whom frailty, multimorbidity, and functional limitations may affect test performance and interpretation [11].

Although previous studies have demonstrated the safety and feasibility of CPET in selected elderly populations, most have focused on specific disease groups, particularly patients with heart failure [12,13]. Consequently, data remain limited for elderly patients with mixed cardiopulmonary comorbidities who are commonly encountered in routine clinical practice. Furthermore, evidence regarding CPET performance in real-world clinical settings, especially for preoperative thoracic surgery assessment and evaluation of unexplained dyspnea, is scarce. This knowledge gap limits the generalizability of existing findings and underscores the need for further studies in broader elderly populations.

Therefore, the present study aimed to evaluate the safety and clinical feasibility of CPET in elderly patients with cardiopulmonary comorbidities.

## 2. Materials and Methods

### 2.1. Study Population

All patients referred for CPET at the Department of Chest Diseases, Ankara University Faculty of Medicine, between January 2013 and January 2025 were retrospectively identified through a computerized database containing demographic characteristics, medical history, medication use, and CPET results. A total of 252 patients underwent CPET during the study period. Seventeen patients were excluded because complete CPET records and/or relevant clinical data were unavailable for retrospective analysis. Consequently, the final study population consisted of 235 unique patients who underwent 235 CPET examinations. No repeated CPET assessments were performed during the study period. Patients were subsequently divided into two groups according to age: those younger than 65 years and those aged 65 years or older.

Patients were categorized as <65 years and ≥65 years because 65 years is the conventional threshold used to define older adults in clinical and epidemiological studies and has been widely adopted in previous investigations evaluating CPET in elderly populations [6].

All patients underwent a standardized pre-test clinical evaluation and were considered suitable for exercise testing by the referring physician and the supervising CPET team. CPET was performed in accordance with contemporary international recommendations [14,15]. Patients with absolute or relative contraindications to exercise testing, including unstable cardiovascular disease, recent myocardial infarction, uncontrolled arrhythmias, acute pulmonary embolism, decompensated heart failure, severe hypoxemia, acute respiratory illness, inability to perform cycle ergometry, severe frailty, or other conditions associated with an unacceptable risk during exercise testing, were not referred for CPET and therefore were not included in the present study. Consequently, all included patients were clinically stable and deemed eligible for CPET at the time of testing.

Our local ethics committee approved the study (ethic approval number: İ05-395-25, on 23 May 2025).

### 2.2. Performance of CPET

In the CPET laboratory pulmonary function tests (spirometry, diffusing lung capacity measurement, lung volumes measurements) and CPET are performed in the same room. The testing environment is supervised by the same pulmonologist. The pulmonologist assists testing personnel with decisions about test protocol and interprets respiratory gas exchange data. All tests are conducted by a 2-person team, consisting of a pulmonologist and pulmonary function technician.

CPET was performed on an electronically braked cycle ergometer using an individualized incremental ramp protocol (CareFusion Germany 234 GmbH). The work-rate increment (5–20 W/min) was selected according to the patient’s age, symptoms, pulmonary function, comorbidities, and estimated exercise capacity, with the aim of achieving symptom-limited maximal exercise of at least 8 min duration. The protocol consisted of a 3-min resting baseline period, followed by 3 min of unloaded cycling as a warm-up. Subsequently, patients performed symptom-limited incremental exercise, after which a 3-min recovery period was completed under continuous monitoring. Throughout the test, electrocardiography, blood pressure, oxygen saturation, and perceived exertion were monitored according to standard laboratory procedures [4].

Maximal exercise effort was determined using established CPET criteria, including a respiratory exchange ratio (RER) ≥ 1.10, achievement of ≥85% of the age-predicted maximal heart rate, development of a VO_2_ plateau when identifiable, and/or high levels of perceived exertion based on Borg dyspnea and fatigue scores [14,15]. Because chronotropic response may be influenced by age and medications, particularly β-blockers, maximal effort was assessed using an integrated interpretation of these parameters rather than a single criterion. Electrocardiography, blood pressure and oxygen saturation were closely monitored during the test. Oxygen uptake (VO_2_), carbon dioxide production (VCO_2_), RER, heart rate reserve (HRR), O_2_ pulse (VO_2_/HR), breath reserve (BR), minute ventilation (VE), and ventilation equivalents (VE/VO_2_, VE/VCO_2_) were determined by CPET. Oxygen saturation, heart rate, and blood pressure were monitored while the dyspnea level was assessed by the Borg dyspnea scale before and at peak exercise tests.

Feasibility was defined as the successful completion of the CPET protocol with acquisition of interpretable physiological data. Feasibility outcomes included completion of the exercise test, exercise duration, achievement of adequate exercise effort according to predefined CPET criteria, and the absence of premature test termination due to adverse events or technical problems. The proportion of patients who completed the protocol and achieved maximal or near-maximal exercise was used as an indicator of clinical feasibility. Because the primary objective of the study was to evaluate CPET applicability in older adults, safety and feasibility outcomes were assessed and analyzed separately.

### 2.3. Identification of Adverse Events

All CPETs were performed in a dedicated laboratory under the supervision of an experienced physician and trained personnel. Continuous 12-lead electrocardiographic monitoring and pulse oximetry were maintained throughout the baseline, exercise, and recovery phases. Blood pressure was measured at baseline and at regular intervals during exercise and recovery, and Borg dyspnea and fatigue scores were recorded during testing. Emergency equipment, including a defibrillator, supplemental oxygen, emergency medications, and a resuscitation cart, was immediately available throughout all procedures.

For the purposes of this study, all CPET reports were reviewed by a single investigator. The occurrence and nature of events during CPET were documented, and in patients experiencing an event, the electronic medical record was further reviewed to obtain a detailed description of the event and its outcome. Events occurring during CPET were categorized as expected exercise-limiting symptoms or clinically significant adverse events. Dyspnea and leg fatigue were considered expected physiological responses to maximal exercise and were not classified as adverse events. However, severe dyspnea resulting in premature test termination was classified as a clinically significant event because it prevented completion of the planned exercise protocol. Clinically significant adverse events included arrhythmia, syncope, chest pain, bronchospasm, desaturation, and severe dyspnea leading to premature test termination. Desaturation was defined as a decrease in SpO_2_ of ≥4% from baseline and/or a nadir SpO_2_ < 88% during exercise. Bronchospasm was defined clinically by the development of wheezing and/or dyspnea requiring bronchodilator administration. Arrhythmias were identified from continuous electrocardiographic monitoring and included rhythm disturbances requiring closer observation or premature test termination. Chest pain events were identified according to contemporaneous physician documentation during testing. Events leading to premature test termination and those requiring medical intervention were recorded separately and analyzed as safety outcomes. No deaths, cardiac arrest, myocardial infarction, or other major complications occurred during testing or within the subsequent three days.

The study was reported in accordance with the Strengthening the Reporting of Observational Studies in Epidemiology (STROBE) recommendations for observational studies [16].

### 2.4. Statistical Analysis

Statistical analyses were performed using the Statistical Package for the Social Sciences (SPSS), version 22.0 (IBM Corp., Armonk, NY, USA). The distribution of continuous variables was assessed using visual methods (histograms and probability plots) and analytical tests (Kolmogorov–Smirnov and Shapiro–Wilk tests). Normally distributed continuous variables were presented as mean ± standard deviation (SD), whereas non-normally distributed and ordinal variables were expressed as median and interquartile range (IQR). Categorical variables were summarized as numbers and percentages.

Comparisons between groups were performed using the independent samples Student’s *t*-test for normally distributed variables and the Mann–Whitney U test for non-normally distributed variables. Categorical variables were compared using the chi-square test or Fisher’s exact test, as appropriate.

Univariable logistic regression analyses were performed to explore factors associated with clinically significant adverse events. Results were reported as odds ratios (OR) with 95% confidence intervals (CI). A *p*-value < 0.05 was considered statistically significant.

## 3. Results

A total of 235 patients who underwent CPET were included in the study. Mean age was 62.3 ± 12.8 years, including 112 (47.6%) patients aged ≥65 years and 35 (14.8%) patients aged ≥75 years. Approximately one-fifth of the population was female. Comorbidities were present in 170 patients, with hypertension being the most common.

Preoperative assessment prior to thoracic surgery was the predominant indication for CPET and accounted for the majority of referrals, particularly among patients aged ≥65 years. Other indications included the evaluation of unexplained dyspnea, pulmonary hypertension, asthma, and exercise intolerance. In the <65 years group, CPET was performed for preoperative assessment in 95 patients, asthma evaluation in 8 patients, and investigation of dyspnea etiology in 22 patients. In the ≥65 years group, 100 patients underwent CPET for preoperative assessment and 12 for evaluation of dyspnea etiology. These findings indicate that the study population predominantly consisted of patients undergoing preoperative physiological assessment in a tertiary respiratory care setting. Seventeen patients (7.2%) were receiving β-blocker therapy at the time of CPET, whereas the majority were not taking cardiovascular medications.

When the groups were compared in terms of comorbidities, diabetes mellitus (DM), hypertension (HT), and cardiovascular disease (CVD) were more frequent in the ≥65 years group compared with the <65 years group, and these differences were statistically significant. In the evaluation of pulmonary function, FEV1/FVC % predicted and FEF_2575_ % predicted values were significantly lower in the ≥65 years group (*p* = 0.001 and *p* = 0.029, respectively) (Table 1).

### 3.1. CPET Feasibility

Adequate exercise effort, defined as achievement of the age-predicted maximal heart rate, was achieved in 77.2% of patients in the <65 years group and 74.1% of patients in the ≥65 years group. When CPET results were compared between groups, the median peak VO_2_ was 19.6 [16.4–22] in the ≥65 years group (*p* < 0.001). This finding is expected, as peak VO_2_ decreases with advancing age. There were no significant differences between the groups in terms of maximal blood pressure or respiratory rate achieved at peak exercise. However, maximal heart rate was significantly lower in the older group (*p* < 0.001) (Table 2).

### 3.2. CPET Safety and Adverse Events

In the ≥65 years group, the exercise phase of the test lasted a mean of 6.37 ± 1.37 min, which was significantly shorter than in the <65 years group.

Clinically significant adverse events included arrhythmia, desaturation, severe dyspnea, chest pain, bronchospasm, and syncope. Of these, desaturation was the only event that did not lead to premature termination of the exercise test. Exercise-induced desaturation occurred in 32 of 123 patients (26.0%) in the <65 years group and in 24 of 112 patients (21.4%) in the ≥65 years group, corresponding to an absolute risk difference of −4.6% (95% CI: −15.6% to 6.4%). The lowest recorded oxygen saturation was 84%, and no desaturation episode required premature termination of the test. Premature termination due to adverse events occurred in 5 of 123 patients (4.1%) in the <65 years group and in 10 of 112 patients (8.9%) in the ≥65 years group, corresponding to an absolute risk difference of 4.9% (95% CI: −1.4% to 11.1%). Although numerically more frequent among elderly patients, premature test termination did not differ significantly between the age groups.

In the ≥65 years group, the test was terminated prematurely in a total of 10 patients due to syncope, dyspnea, bronchospasm, chest pain, or arrhythmia. In all three patients who developed arrhythmia, frequent extrasystoles were observed, leading to early termination of the test. The patient who developed bronchospasm was treated with a short-acting bronchodilator. No serious adverse events, including cardiac arrest, myocardial infarction, or death, were observed during CPET or within the subsequent three days. Compared with the <65 years group, there was no statistically significant difference in the incidence of adverse events during the test (Table 2).

### 3.3. Predictors of Adverse Events

Logistic regression analysis was performed in 112 elderly patients, including 10 patients with clinically significant adverse events and 102 patients without adverse events. In the univariable logistic regression analysis, smoking history (OR: 4.90, 95% CI: 1.46–16.42, *p* = 0.010), lower FEV1 % predicted (OR: 0.96, 95% CI: 0.94–0.99, *p* = 0.020), and lower FEV1/FVC ratio (OR: 0.90, 95% CI: 0.84–0.96, *p* = 0.001) were significantly associated with clinically significant adverse events. No significant associations were observed for sex, BMI, diabetes mellitus, hypertension, cardiovascular disease, pulmonary hypertension, COPD, asthma, FVC, DLCO, peak VO_2_, or exercise testing duration (all *p* > 0.05). Given the small number of adverse events, the available data were insufficient to support a robust multivariable logistic regression analysis according to commonly recommended event-per-variable considerations. Therefore, we elected to restrict the analysis to univariable models to minimize the risk of overfitting and unreliable effect estimates. Accordingly, the observed association between FEV1/FVC ratio and adverse events should be interpreted as an unadjusted finding that warrants confirmation in larger studies with sufficient outcome events to permit appropriate multivariable adjustment (Table 3).

## 4. Discussion

Our findings demonstrate that no major complications were observed in this cohort of elderly patients undergoing CPET. In addition to safety, our findings support the clinical feasibility of CPET in elderly patients. Most older adults were able to complete symptom-limited exercise testing and provide clinically interpretable physiological data. Although premature test termination occurred in 10 patients in the ≥65-year group, the majority of examinations yielded adequate exercise effort and clinically useful CPET parameters. The observed exercise duration and effort indices suggest that age alone did not preclude successful test completion. These findings are particularly relevant because concerns regarding frailty, exercise intolerance, and reduced physiological reserve frequently limit referral for CPET in older adults. Our results indicate that, when performed under appropriate supervision and with individualized protocols, CPET can be successfully implemented in routine clinical practice in elderly patients with cardiopulmonary comorbidities [4]. Interpretation of exercise effort was based on multiple CPET parameters, including RER and exercise performance indices, rather than heart rate criteria alone, since β-blocker therapy and age-related chronotropic limitations may affect the achievement of age-predicted maximal heart rate in older adults. Importantly, the majority of patients in our cohort, particularly those aged ≥65 years, were referred for preoperative thoracic surgery evaluation in a tertiary respiratory care center. Therefore, our findings may be most applicable to older surgical candidates undergoing preoperative functional and physiological assessment in a tertiary respiratory care setting and should not necessarily be generalized to all elderly patients with cardiopulmonary disease.

In our cohort, most elderly patients were asymptomatic or only mildly symptomatic despite a high burden of comorbidities. Consequently, comparisons with previous CPET safety studies should be interpreted with caution, as many of those investigations were conducted in selected cardiovascular populations, whereas our cohort reflected a real-world clinical population with mixed cardiopulmonary comorbidities and a substantial proportion of patients undergoing preoperative thoracic surgery assessment. Notably, 80.4% of patients in the ≥65 years group had at least one comorbid condition. Previous studies evaluating CPET safety have primarily focused on populations with cardiovascular disease [6,17,18,19]. In contrast, our study included not only patients with cardiovascular disorders but also those with respiratory comorbidities such as COPD, asthma, and pulmonary hypertension, as well as systemic diseases including diabetes mellitus. The higher burden of comorbidities in older patients should be considered when interpreting age-group comparisons. The high prevalence of diabetes mellitus, hypertension, cardiovascular disease, and chronic respiratory disorders in our cohort may further underscore the potential interplay between cardiometabolic and pulmonary health in older adults. The coexistence of cardiometabolic and respiratory comorbidities may influence exercise performance through a variety of physiological mechanisms that could not be fully explored within the scope of the present study [20,21]. Recent evidence has suggested that cardiometabolic and chronic respiratory diseases may be linked through shared biological pathways that could contribute to functional outcomes in ageing populations [21]. Although these mechanisms were not specifically evaluated in the present study, they should be considered when interpreting CPET findings in elderly patients with multiple comorbidities. In addition, unlike many previous studies, spirometric and diffusion capacity measurements were evaluated together with CPET parameters.

The present findings should also be interpreted in the context of contemporary CPET literature and guideline recommendations. Previous studies have demonstrated a low incidence of serious adverse events during CPET in both high-risk cardiovascular populations and elderly individuals, supporting its role as a safe and informative physiological assessment when performed under appropriate supervision. More recently, the Study of Muscle, Mobility and Aging (SOMMA) demonstrated the feasibility of standardized CPET protocols in community-dwelling older adults, further supporting the applicability of CPET in ageing populations [10]. In addition, the ERS technical standards emphasize the importance of standardized testing protocols, appropriate patient selection, and continuous physiological monitoring to ensure test quality and safety [4]. Given that preoperative thoracic surgery evaluation represented the most common indication for CPET in our cohort, our findings are also aligned with the recent ERS/ESTS guideline on lung cancer treatment, which recommends CPET as part of the preoperative physiological evaluation of patients being considered for lung cancer surgery [3]. Consistent with this recommendation, CPET was routinely used in our center to support the functional and physiological assessment of surgical candidates. Our results suggest that CPET can be safely and feasibly performed in older adults undergoing such evaluations when appropriate patient selection and medical supervision are ensured [3].

Adverse events during CPET are uncommon when testing is performed under appropriate supervision [4]. In the largest study assessing CPET safety, 5060 high-risk patients with cardiovascular disease, including 196 patients with pulmonary arterial hypertension, underwent CPET, with an overall adverse event rate of only 0.16% and no reported mortality [19]. Importantly, 14% of that cohort consisted of patients aged ≥75 years. Sustained ventricular tachycardia was the most frequently observed complication and occurred in only six patients. These findings further suggest that CPET may be performed with a low rate of serious complications in carefully selected elderly individuals with significant cardiac disease.

Similarly, in a cohort of 347 patients with aortic stenosis (mean age 66 ± 14.1 years), CPET was safely performed without major complications despite the high-risk nature of the study population [17]. Although current guidelines recommend caution regarding CPET in aortic stenosis unless clinically justified, exercise testing may still be required for functional assessment and perioperative evaluation [22]. Furthermore, another study involving 227 elderly patients with heart failure (mean age 76 years) demonstrated that CPET could be safely conducted with a low complication risk [18].

Our findings are also in agreement with the prospective SOMMA cohort, which demonstrated that CPET can be safely and successfully performed in adults aged 70 years and older, including those with multimorbidity and frailty. While the SOMMA study primarily involved community-dwelling older adults undergoing treadmill-based CPET in a research setting, our cohort reflects a real-world tertiary respiratory care population with substantial cardiopulmonary comorbidity and a predominance of patients referred for preoperative thoracic evaluation [10]. Despite these differences, both studies support the feasibility and safety of CPET in older adults when appropriate supervision and standardized protocols are applied.

A Korean study evaluating 1485 patients with cardiovascular disease (mean age 71.6 ± 4.7 years) reported adverse events in only three patients, all of whom developed ventricular arrhythmias during testing [6]. Unlike that study, our cohort was not restricted to cardiovascular disease and also included patients with pulmonary comorbidities that could potentially affect CPET performance and safety. Although diffusion capacity parameters were similar between age groups, no increase in CPET-related adverse events, particularly exercise-induced desaturation, was observed compared with younger patients. Lower FEV1/FVC ratio and lower FEV1 % predicted were associated with adverse events during CPET. This finding may reflect the impact of underlying airflow limitation on exercise tolerance and physiological responses during exertion. Smoking history was also associated with clinically significant adverse events in the univariable analysis, possibly reflecting the cumulative effects of smoking-related cardiopulmonary impairment. However, given the limited number of clinically significant adverse events observed in the present study and the absence of multivariable adjustment, these findings should be interpreted cautiously and require confirmation in larger prospective studies. In contrast, despite similar COPD prevalence between age groups, COPD itself was not associated with increased adverse events during CPET. Although no increased risk was observed in patients with COPD in our cohort, this finding should be interpreted cautiously given the limited number of events and the lack of disease-specific subgroup analyses. Exercise-induced desaturation was the most frequently observed clinically significant event in our cohort. However, no desaturation episode resulted in premature test termination, and the lowest recorded oxygen saturation was 84%. In elderly patients with underlying respiratory disease, exercise-induced desaturation may reflect impaired gas exchange and reduced pulmonary reserve rather than an unexpected complication of testing [23,24,25]. Although desaturation alone does not necessarily mandate long-term oxygen therapy, its presence may prompt further clinical evaluation, including assessment of gas exchange abnormalities, disease severity, and the need for additional monitoring during exertion. These findings also highlight the importance of continuous pulse oximetry during CPET, particularly in older adults with respiratory comorbidities [4].

CPET is also a valuable noninvasive modality in patients with suspected pulmonary hypertension (PH), providing important information for differential diagnosis, risk stratification, and treatment planning. Previous studies have demonstrated that combining specific CPET parameters may improve the detection of PH cases that could otherwise be missed by echocardiography alone [26,27]. In addition, CPET may assist in distinguishing PH from other common causes of exertional dyspnea, including heart failure and COPD. Exercise-induced desaturation is considered more suggestive of PH, whereas oscillatory ventilation during exercise is more commonly associated with heart failure [28,29,30,31]. Current guidelines further recommend CPET for baseline and follow-up risk assessment in patients with Group 1 PH, with peak $\dot{V}$O_2_, percent predicted $\dot{V}$O_2_, and $\dot{V}$E/$\dot{V}$CO_2_ slope recognized as major prognostic indicators [32,33,34]. Serial CPET assessments may also provide valuable information regarding treatment response in high-risk populations such as patients with connective tissue diseases or prior pulmonary embolism. However, data regarding CPET safety in elderly patients with PH remain limited. In our cohort, although no patients had primary pulmonary hypertension, individuals with elevated pulmonary artery pressure estimated by echocardiography (>40 mmHg) underwent CPET without major complications, suggesting that the test may be safely applied in this subgroup under appropriate supervision.

This study has several limitations. First, this was a retrospective, single-center study conducted at a tertiary referral center, which may limit the generalizability of the findings. Second, adverse events were identified through retrospective review of CPET reports and medical records; therefore, minor events may have been underreported despite systematic review of available documentation. Third, referral practices and pre-test clinical screening may have introduced selection bias, as patients with absolute or relative contraindications to CPET were not referred for testing. Consequently, the observed safety profile may not be representative of all elderly patients with cardiopulmonary disease. Fourth, all patients underwent CPET using a single-cycle ergometer protocol, limiting extrapolation of the findings to other exercise modalities and testing protocols. Fifth, several geriatric and functional parameters were not systematically assessed, including frailty status, physical activity levels, sarcopenia-related measures, peripheral muscle strength, ASA classification, and baseline functional independence. These factors may substantially influence exercise capacity, CPET performance, and premature test termination in older adults and therefore should be considered when interpreting the feasibility findings. In addition, several CPET-derived physiological variables, including anaerobic threshold, VE/VCO_2_ slope, breathing reserve, and ventilatory limitation patterns, were not consistently available for all participants and therefore could not be included in the analysis. As a result, the mechanisms underlying exercise limitation and adverse events could not be fully characterized. Sixth, the study was not specifically designed or powered to perform disease-specific subgroup analyses. In addition, the limited number of clinically significant adverse events reduced the statistical power available for multivariable analyses and may have limited our ability to evaluate adjusted associations between potential predictors and adverse events. Consequently, the impact of individual comorbidities, such as COPD, asthma, pulmonary hypertension, and cardiovascular disease, on CPET safety and feasibility could not be evaluated in detail. Finally, follow-up was limited to events occurring during testing and the immediate post-test period; therefore, delayed clinical outcomes and long-term consequences could not be evaluated. Future prospective multicenter studies with larger sample sizes and standardized outcome assessment are warranted to further define the safety and feasibility of CPET in elderly patients. Furthermore, the study was not powered to establish equivalence between age groups or to perform robust analyses in the subgroup of patients aged ≥75 years.

Despite these limitations, our study also has important strengths. To our knowledge, it represents one of the larger cohorts specifically evaluating CPET safety in elderly patients with substantial cardiopulmonary comorbidity burden. In addition, pulmonary function parameters, including spirometry and diffusion capacity measurements, were comprehensively assessed, allowing a more detailed interpretation of exercise performance and adverse events.

Overall, no major complications were observed during CPET in this cohort of elderly patients, including those with substantial cardiopulmonary comorbidity. When performed under appropriate supervision, CPET appears to be a feasible and clinically valuable tool for functional assessment in elderly patients. Nevertheless, larger prospective multicenter studies are required to further evaluate its safety profile and detect rare adverse events.

In conclusion, CPET was feasible in the majority of elderly patients with cardiopulmonary comorbidities, with most individuals successfully completing testing and providing clinically interpretable physiological data. Although premature test termination occurred in a small proportion of patients, no major complications were observed during testing or the immediate follow-up period. These findings suggest that, when performed under appropriate supervision and with careful patient selection, CPET may represent a practical and feasible tool for functional assessment in older adults. Given the retrospective single-center design and the limited ability to detect rare adverse events, larger prospective multicenter studies are warranted to further define the safety and feasibility of CPET in this population.

From a practical perspective, several take-home messages emerge from our findings. First, advanced age alone should not be considered a barrier to CPET referral when appropriate patient selection and medical supervision are ensured. Second, most elderly patients were able to successfully complete testing and provide clinically interpretable physiological data, supporting the feasibility of CPET in routine clinical practice. Finally, CPET may provide valuable physiological information during the evaluation of unexplained exercise intolerance and functional limitation in older adults with cardiopulmonary comorbidities.

## Figures and Tables

**Table 1:** Comparison of demographic, clinical, and functional variables between groups.

	<65 Years *n*:123	≥65 Years *n*:112	*p* Value
Age, years ± SD	53.42 ± 11.16	72.04 ± 5.04	<0.001
Gender, male, *n* (%)	91 (74)	96 (85.7)	0.035
BMI, kg/m^2^ ± SD	25.40 ± 5.56	25.99 ± 4.31	0.366
Smoking history, *n* (%)			0.101
Current	37 (30.1)	36 (32.1)	
Former	51 (41.5)	57 (50.9)	
Never	35 (28.5)	19 (17)	
Comorbidities, *n* (%)			
DM	10 (8.1)	20 (17.9)	0.031
HT	28 (22.8)	47 (42)	0.002
CVD	11 (8.9)	37 (33)	<0.001
Pulmonary HT	6 (4.9)	10 (8.9)	0.301
COPD	24 (19.5)	24 (21.4)	0.748
Asthma	13 (10.6)	7 (6.3)	0.253
ILD	5 (4.1)	0	0.061
β-Blockers, *n* (%)	7 (5.6)	10 (8.9)	0.336
CPET indication, *n* (%)			
Preoperative evaluation	95 (77.2)	100 (89.3)	0.013
Diagnosis for asthma	8 (6.5)	0	<0.001
Dyspnea etiology	22 (17.8)	12 (10.7)	0.043
Spirometry, median [IQR]			
FEV1, predicted %	77 [63–90.5]	73 [62–88]	0.280
FVC, predicted %	92 [74.5–102]	87 [78–99]	0.890
FEV1/FVC, %	70 [61–79]	65 [59–72]	0.001
FEF_2575_, predicted %	45.5 [28.5–69]	33 [24–54.7]	0.029
Diffusing capacity, median [IQR]			
DLCO, predicted %	60 [47–74]	58.5 [50–70]	0.878
DLCO/VA, predicted %	81 [63–91]	77 [64.2–88]	0.693

SD: standard deviation, *n*: number, kg/m^2^: kilogram/meter^2^, IQR: interquartile range, FEV1: forced expiratory volume in first second, FVC: forced vital capacity, FEF_2575_: forced expiratory flow between 25% and 75% of forced vital Capacity, DLCO: diffusing lung capacity for cabon monoxide, VA: volume alveolar, DM: diabetes mellitus, HT: hypertension, CVD: cardiovascular disease, COPD: chronic obstructive pulmonary disease, ILD: interstitial lung disease.

**Table 2:** Comparison of CPET performance, feasibility outcomes, and safety events between age groups.

	<65 Years *n*:123	≥65 Years *n*:112	*p* Value
Peak VO_2_, mL/kg/min, median [IQR]	22.8 [20–26]	19.6 [16.4–22]	<0.001
Percent of predicted peak VO_2_%, median [IQR]	74 [66–89]	90.5 [75–104]	<0.001
Evaluation of maximal exercise			
RER, mean ± SD	1.03 ± 0.18	1.02 ± 0.19	0.543
Achievement of the age-predicted max. heart rate, *n* (%)	95 (77.2)	83 (74.1)	0.648
High levels of perceived exertion based on Borg, *n* (%)	21 (17.1)	64 (57.1)	<0.001
Achievement of peak VO_2_, *n* (%)	41 (33.3)	68 (60.7)	<0.001
At peak exercise			
Heart rate, bpm, median [IQR]	147 [136–157.25]	133 [121–145]	<0.001
Respiratory rate/min, median [IQR]	37.5 [33–44.25]	36 [33–42]	0.497
Systolic blood pressure, mmHg, median [IQR]	170 [140.5–194.75]	170 [155.2–190.0]	0.511
Diastolic blood pressure, mmHg, median [IQR]	90 [76.25–100]	89.5 [77.2–97.7]	0.659
Testing time on exercise, min, mean ± SD	6.88 ± 1.21	6.37 ± 1.37	0.004
Early termination due to adverse events, *n* (%)	5 (4)	10 (8.9)	0.118
Adverse events leading to early termination, *n* (%)			
Arrhythmia	1 (0.8)	3 (2.6)	0.349
Chest pain	1 (0.8)	1 (0.9)	1.000
Bronchospasm	1 (0.8)	1 (0.9)	1.000
Syncope	1 (0.8)	2 (1.8)	0.606
Severe dyspnea	1 (0.8)	3 (2.6)	0.350

SD: standard deviation, *n*: number, IQR: interquartile range, mL/kg/min: milliliter/kilogram/minute, VO_2_: peak oxygen uptake; bpm: beats per minute.

**Table 3:** Evaluation of factors affecting the occurrence of adverse events in elderly patients using Binary Logistic Regression analysis.

	Univariate Logistic Regression Analysis	
	Odds Ratio (95% Cl)	*p* Value
Male gender	3.16 (0.84–11.82)	0.087
BMI	0.91 (0.80–1.04)	0.202
Smoking history	4.90 (1.46–16.42)	0.010
DM	0.77 (0.19–3.05)	0.710
HT	1.35 (0.42–4.32)	0.613
CVD	0.87 (0.27–2.81)	0.820
Pulmonary HT	0.28 (0.06–1.25)	0.096
COPD	1.73 (0.36–8.35)	0.491
Asthma	0.39 (0.08–1.88)	0.245
FVC, predicted %	0.99 (0.95–1.02)	0.594
FEV1, predicted %	0.96 (0.94–0.99)	0.020
FEV1/FVC, %	0.90 (0.84–0.96)	0.001
DLCO, predicted %	0.98 (0.94–1.01)	0.330
Peak VO_2_, mL/kg/min	1.06 (0.93–1.21)	0.321
Testing time on exercise	1.11 (0.72–1.69)	0.627

BMI: body mass index, DM: diabetes mellitus, HT: hypertension, CVD: cardiovascular disease, COPD: chronic obstructive pulmonary disease, FVC: forced vital capacity, FEV1: forced expiratory volume in first second, DLCO: diffusing lung capacity for carbon monoxide, VO_2_: peak oxygen uptake.

## Data Availability

The datasets analyzed during the current study are available from the corresponding author on reasonable request.

## Abbreviations

ASA	American Society of Anesthesiologists
BMI	Body Mass Index
BR	Breathing Reserve
CI	Confidence Interval
COPD	Chronic Obstructive Pulmonary Disease
CPET	Cardiopulmonary Exercise Testing
CVD	Cardiovascular Disease
DLCO	Diffusing Capacity of the Lung for Carbon Monoxide
DM	Diabetes Mellitus
ERS	European Respiratory Society
ESTS	European Society of Thoracic Surgeons
FEF_2575_	Forced Expiratory Flow Between 25% and 75% of Forced Vital Capacity
FEV1	Forced Expiratory Volume in One Second
FVC	Forced Vital Capacity
HRR	Heart Rate Reserve
HT	Hypertension
ILD	Interstitial Lung Disease
IQR	Interquartile Range
OR	Odds Ratio
PH	Pulmonary Hypertension
RER	Respiratory Exchange Ratio
SD	Standard Deviation
SpO_2_	Peripheral Oxygen Saturation
STROBE	Strengthening the Reporting of Observational Studies in Epidemiology
VA	Alveolar Volume
VCO_2_	Carbon Dioxide Production
VE	Minute Ventilation
VO_2_	Oxygen Uptake
